# Supplementary material for: A method for multiplexed full-length single-molecule sequencing of the human mitochondrial genome
Source: Nat Commun. 2022 Oct 6;13:5902. doi: 10.1038/s41467-022-33530-3 (PMC9537161; doi:10.1038/s41467-022-33530-3)

A method for multiplexed full-length single-molecule sequencing of the human mitochondrial genome

(Supplementary Information)

Ieva Keraite[1], Philipp Becker[1‡], Davide Canevazzi[1], Cristina Frias-López[1], Marc Dabad[1], Raúl Tonda-Hernandez[1], Ida Paramonov[1], Matthew John Ingham[1], Isabelle Brun-Heath[2,3], Jordi Leno[4,5], Anna Abulí[4,5], Elena Garcia-Arumí[4,6,7], Simon Charles Heath[1,8], Marta Gut[1,8*] & Ivo Glynne Gut[1,8]*

[1] CNAG-CRG, Centre for Genomic Regulation (CRG), The Barcelona Institute of Science and Technology (BIST), Barcelona, Spain

[2] Institute for Research in Biomedicine (IRB Barcelona) - The Barcelona Institute of Science and Technology (BIST), Barcelona, Spain

[3] Joint IRB-BSC Program in Computational Biology, Barcelona, Spain

[4] Department of Clinical and Molecular Genetics and Rare Disease, Hospital Universitari Vall d'Hebron, Barcelona, Spain

[5] Medicine Genetics Group, VHIR, Hospital Universitari Vall d'Hebron, Barcelona, Spain

[6] Research Group on Neuromuscular and Mitochondrial Disorders, VHIR, Hospital Universitari Vall d'Hebron, Barcelona, Spain

[7] Centro de Investigación Biomédica en Red de Enfermedades Raras (CIBERER), Instituto de Salud Carlos III, Barcelona, Spain

[8] Universitat Pompeu Fabra, Barcelona, Spain

[‡] Present address: Qiagen, Hilden, Germany

*All correspondence should be addressed to:
marta.gut@cnag.crg.eu
ivo.gut@cnag.crg.eu

Supplementary Note 1

Guide RNA (gRNA) selection and multiplexing strategy. We used the Geneious Prime 2021.2.2 tool to identify 2,198 potential guides, of which 428 were complementary to the light strand (LS) and 1,770 to the heavy strand (HS). Once filtered according to the Doench (2014) Activity Score[1] and disregarding all sequences that have a score of <0.3, we obtained 132 LS and 397 HS guide sequences. This classification only informs on the level of cleavage efficiency based on the observations of guide RNA sequence features. It is speculated that there are differences between biochemical and cell-based Cas9 cleavage due to RNA folding, stability, complex formation[2], and available off-target sequences. In our approach for designing synthetic CRISPR RNA (crRNA) sequences the most important factor was a low number of off-targets resulting in stringent enrichment of mtDNA molecules (avoiding hypervariable regions and well-described variants). We generally used two guides in two separate aliquots for each sample, as shown in Fig. 1. The guides are located approximately 8 kb from each other, one targets the LS and the other the HS, in order to produce coverage data over the cut sites and to identify areas where cutting efficiency is reduced due to mtDNA alterations. In more complex situations, when analysing deletions, it is possible to use more than two guides in a corresponding number of aliquots per sample located in strategically selected positions. We designed and tested eight gRNAs spanning the entire mtDNA sequence considering hypervariable regions and major pathogenic variants, to multiplex up to four samples (Supplementary Table 1).

Supplementary Note 2

Implementation and optimization of Cas9-mtDNA-enrichment. First, to validate the method and to demonstrate the preferential sequencing of the full-length mtDNA molecule with high output we used a gDNA sample in two aliquots with one pair of gRNAs. Here we used

high integrity gDNA extracted from the human embryonic kidney HEK293 cell line to enrich for intact full-length mtDNA and to demonstrate reliable demultiplexing. As a control, we added three blank (without gDNA) samples incubated each with a different pair of gRNAs. We achieved a coverage of the targeted region exceeding 5,000× (Supplementary Fig. 1a). We also observed an extremely low number of reads (<0.02%) with non-intended cuts from the blank reactions, confirming our approach for reliable demultiplexing of multiple pooled samples (Supplementary Data 2). Next, we focused on increasing the data output and enrichment. It was indicated that the Cas9 complex stays bound to the 5’ end of the guide RNA[3]. In our case, meaning the Cas9 complex stays bound to the end of the linearized mtDNA molecules. This may obstruct transition of the DNA molecule through the nanopore and reduce sequencing yield[4]. Therefore, we used Proteinase K to digest the Cas9 nuclease. By introducing the Proteinase K digest, we achieved a 2-fold increase of the full-length reads fraction mapping to the mtDNA reference (Supplementary Fig. 1b).

To reduce the nuclear DNA fraction in the starting material and to increase the final enrichment of the circular mtDNA molecules we introduced an Exonuclease V treatment upstream. In this strategy we used high integrity gDNA extracted from HEK293, Capan-2, A549, SH-SY5Y cell lines (Supplementary Fig. 1c) and we achieved a mean increase of the mtDNA fraction from 27% to 49% of total reads on a GridIon R10.4 flow cell (Supplementary Data 3).

With the addition of Proteinase K and Exonuclease V steps, we achieved a high sequencing depth for single and multiplexed samples (Supplementary Fig. 1d). However, performing DNA quality control upfront of the gDNA processing is very important to decide whether introducing the Exonuclease V treatment step will be useful. Inclusion of the Exonuclease V treatment step for low integrity gDNA samples is counterproductive as it leads to digestion of fragmented mtDNA molecules.

a

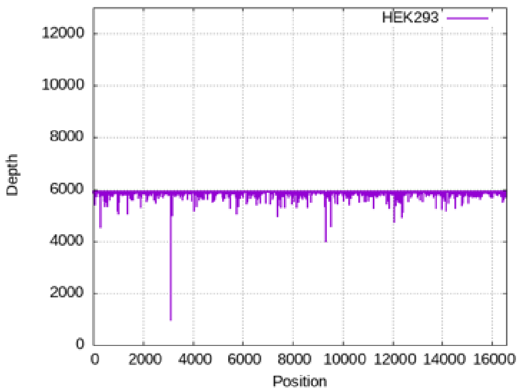

b

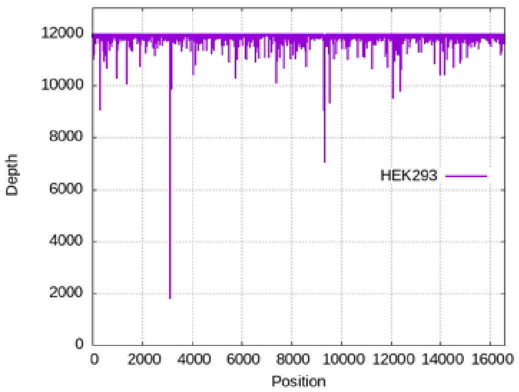

c

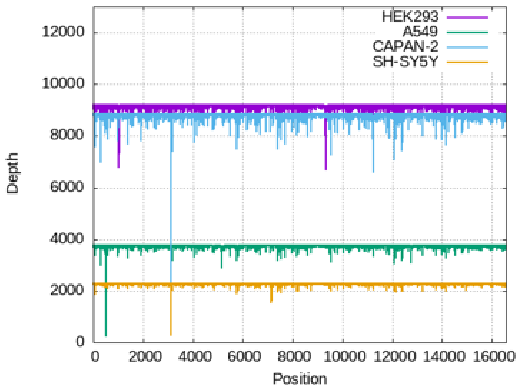

d

Cas9-mtDNA enrichment ONT sequencing	Cell lines		
	Single (Prot K-)*	Single (Prot K+)*	Multiplex**
Mapped reads	61297	65690	109829
Full-length mtDNA	5928	11980	24935
All mtDNA reads	23515	32769	59656
mtDNA read %	38.4%	49.9%	54%

* HEK293 cell line

** Multiplexed HEK293, A549, Capan-2, SH-SY5Y cell lines

Supplementary Figure 1: Sequencing depth data of simplex and multiplex Cas9-mtDNA-enrichment experiments. Sequencing depth plots of full-length mtDNA reads (as analysed with the ‘**Both**’ strategy) in three separate cell line mtDNA enrichment experiments: (**a**) a single HEK293 DNA sample sequenced on a GridIon R10.4 flow cell without Proteinase K treatment; (**b**) a single HEK293 DNA sample sequenced on a GridIon R10.4 flow cell with Proteinase K treatment; (**c**) DNA of 4 cell lines (HEK293, A549, Capan-2, SH-SY5Y) treated with Exonuclease V and Proteinase K, and multiplexed on one GridIon R10.4 flow cell. (**d**) summary showing total read count mapped to the human whole genome reference GRCh38, full-length mtDNA read count, and the percentage of full-length mtDNA reads. Source data are provided as a Source Data file.

Supplementary Note 3

Multiplexed sequencing assay with different GridIon flow cells. We applied our method to four cell line DNA samples (HEK293, Capan-2, A549, SH-SY5Y) utilizing two gRNAs per sample for multiplexing, Proteinase K and Exonuclease V. The library of the pooled samples was prepared with the Q20+ chemistry (Kit 12 chemistry) and run on different GridIon flow cell versions - R9.4.1, R10.3 and R10.4 (Supplementary Data 4). The percentage of on-target

reads mapping to mtDNA reached >60% on R9.4.1 flow cells, 57% on R10.3 and 52% on R10.4 flow cells. The coverage of the full-length mtDNA in the library pool ranged from 4,500× to 11,559× on the R9.4.1 flow cell vs. 2,002× and 5,040× on the R10.3 flow cell after demultiplexing. Comparing the pooled library on R9.4.1 vs. R10.4, coverage ranged from 1,739× to 8,318× vs. 2,347× to 9,444×, respectively. In this comparison, the lowest output as expected was in the R10.3 flow cell, while the yield obtained with R9.4.1 and R10.4 flow cells was comparable.

Supplementary Note 4

Remapping to the custom reference. As described in the Methods, a custom reference sequence was made for each cut site (Supplementary Fig. 2). Use of the custom references allows full and partial length reads to map uniquely to the reference without requiring a split across the reference origin.

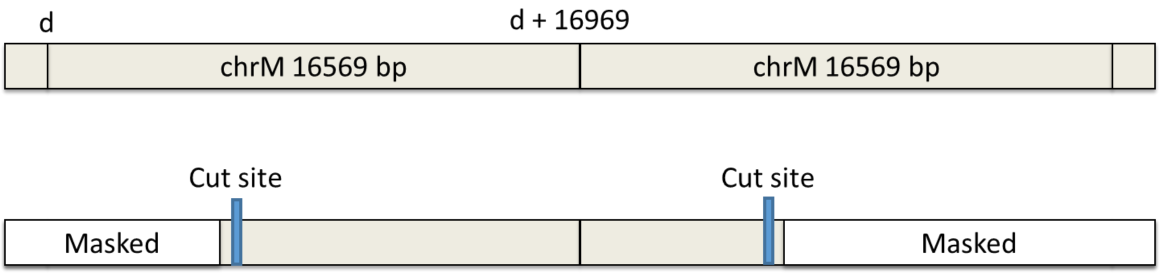

Supplementary Figure 2: Custom references of chrM. Top: general form of custom references showing the 2 tandem copies of the mitochondrial sequence. Bottom: a cut site specific reference showing the masking of bases lying outside of the two locations of the cut site on the reference.

Supplementary Note 5

Validation of the variant calling pipeline and assessment of multiplexing bleed-through.

To validate the multiplexing strategy reliability, we compared the nanopore sequencing genotype data from four separate R10.4 flow cells to Illumina short-read sequencing of mtDNA enriched by long-range PCR (lrPCR) in gDNA samples from the four cell lines (HEK293, Capan-2, A549, SH-SY5Y). A blacklist of mtDNA reference sequence positions was established for automatic filtering from the variant calling pipeline (Supplementary Data 5a) including sites that gave inconsistent calls across replicates or that were called as heteroplasmic across most of the samples.

The GATK mitochondrial calling pipeline was then used to call variants from the Illumina data of the four cell line gDNA samples. Across the mtDNA genome, 45 positions (including one 2 bp deletion) were identified by GATK where at least one sample had an alternative allele in homoplasmy (Supplementary Data 5b). The same 45 positions were also identified as homoplasmic variants from the ONT sequencing data, and the variant calls were completely concordant across all four flow cells as well as with the Illumina short-read sequencing results. Additionally, 26 positions were called from the Illumina data as being heteroplasmic with estimated alternate allele frequencies ≥ 0.5%, none of the putative heteroplasmic variants were shared across multiple samples (Supplementary Data 5c). All 26 sites were also identified (with the same alternate allele) using the ONT data, although for some sites the variant call was indicated as being of low quality with one or more of the flow cells. Estimates of alternate allele frequencies from Illumina and ONT were highly concordant (Supplementary Fig. 3).

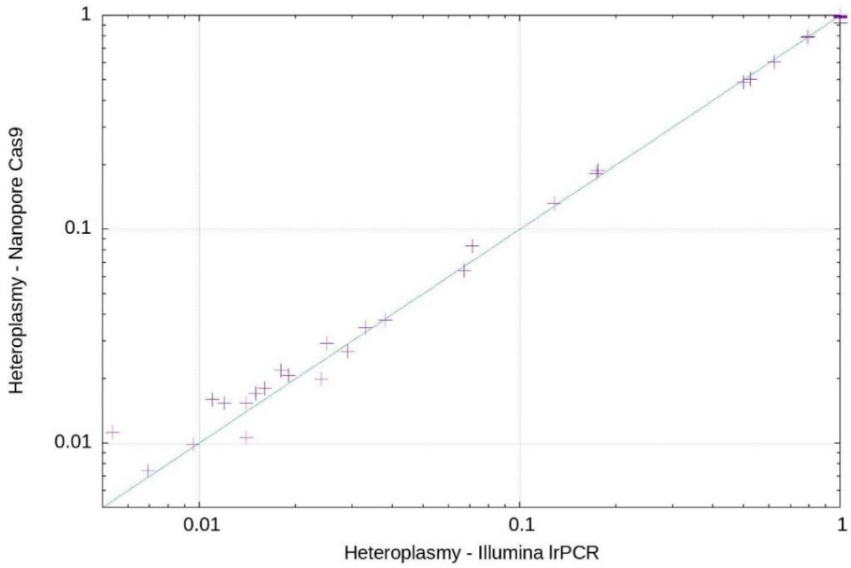

Supplementary Figure 3: Correlation between the mtDNA heteroplasmy frequencies in cell lines measured by lrPCR Illumina and Cas9-mtDNA-enrichment ONT sequencing. Source data are provided as a Source Data file.

We can assess the performance of the calling using the *sensitivity* (the proportion of true calls that are detected by our method) and the *precision* (the proportion of variant calls that are true). The variant allele frequency (VAF) has to be taken into account when assessing the performance. For example, if we only look at variants where VAF ≥ 5% then both sensitivity and precision are 1 for all 4 flow cells. This means that the ONT and Illumina derived results are 100% concordant where VAF ⩾ 5%. If, however, we look only at variants with heteroplasmy < 5% the sensitivity varies from 0.63 – 0.89 and the precision from 0.83 – 1. The variability in the estimates across flow cells comes to a large part from differences in coverage. Two of the flow cells had the Exonuclease V treatment, while the other two did not; the Exonuclease V treated flow cells had over twice as many reads mapping to the mtDNA than the other two flow cells. The two flow cells with the lowest coverage have an average sensitivity and precision of 0.66 and 0.88, while the corresponding figures for the two flow cells with highest coverage are 0.88 and 0.94. We therefore pooled the results across all 4 flow cells to get an average sensitivity of 0.80 and precision of 0.91 for low VAF variants

(heteroplasmy from 0.5% - 5%), and an average sensitivity and precision of 0.88 and 0.95 for all heteroplasmic variants with VAF ⩾ 0.5%.

To investigate to what extent the high accuracy achieved depends on using the R10.4 flow cells and Q20+ chemistry, we also ran the same 4 samples on a R9.4.1 flow cell with pre-Q20+ chemistry (Kit 10), and performed the same analyses as were done for the Q20+ chemistry paired with the R10.4 flow cells (Supplementary Data 3). In this case, the sensitivity and specificity for the low frequency variants were 0.75 and 0.92, while for all heteroplasmic variants they were 0.81 and 0.91. These results are within the ranges obtained from the analyses of the Q20+ chemistry with the R10.4 flow cells, thus demonstrating that the analysis pipeline can still perform well using data produced using the pre-Q20+ chemistry.

Three high frequency heteroplasmic variants were identified in the SH-SY5Y cell line from both the Illumina and ONT data (m.1217G>A, m.15490C>A, m.15961G>T) with estimated frequencies from ONT of 19.2%, 17.8% and 49.2%, respectively. Using the full-length mtDNA reads, we were able to determine all of the haplotypes of the 3 variants present in this sample. Out of the 8 possible haplotypes, GCT, GCG and AAG were present at high frequency (48.6%, 32.0% and 18.4%, respectively) with the other 5 haplotypes making up the remaining 1%. In short-read Illumina sequencing we confirmed the SNVs frequencies but it was only possible to resolve the phase of the two variants positioned nearby each other (m.15490, m.15961), due to the inherent limitations of short-read sequencing (Supplementary Fig 4).

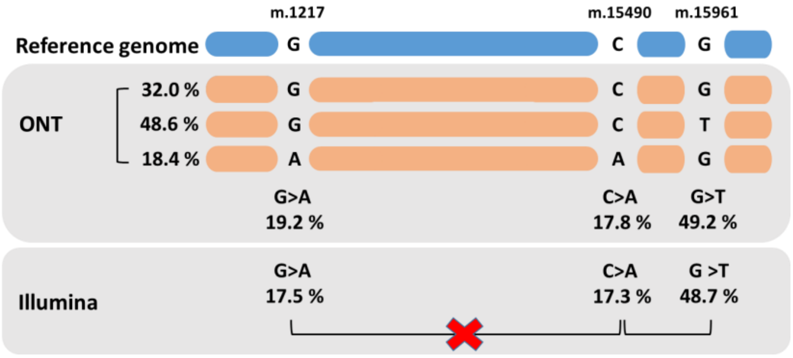

Supplementary Figure 4: mtDNA SNVs phasing with long- and short-read sequencing. Visual representation of resolved SH-SY5Y cell line mtDNA haplotypes of the distant variants by nanopore sequencing. Using Illumina data only, the m.1217-m.15490 phased SNVs stay unresolved.

Supplementary Note 6

Quality control of clinical samples by Femto Pulse. High-resolution DNA sizing was performed as part of the clinical gDNA sample quality control (Supplementary Fig. 5). The integrity is visualized by the Femto Pulse (Agilent Technologies, Agilent Genomic DNA 165 kb kit) traces and expressed by the DNA quality number (DQN). The DQN calculation is based on the fraction of the total measured concentration of the sample that lies above the specified size threshold. DQN scores samples on a scale from 0 to 10, 0 indicating that none of the sample exceeds the threshold and 10 indicates 100 % of the sample lies above the threshold value. The Femto Pulse traces manifested better integrity profiles and higher DQN for the blood gDNA samples, while the samples from buccal swab (AW6491, AW6492) and urine (AW6494) showed more degradation that subsequently, in the Cas9-mtDNA-enrichment sequencing, could be directly related to a lower recovery of full-length mtDNA reads.

a

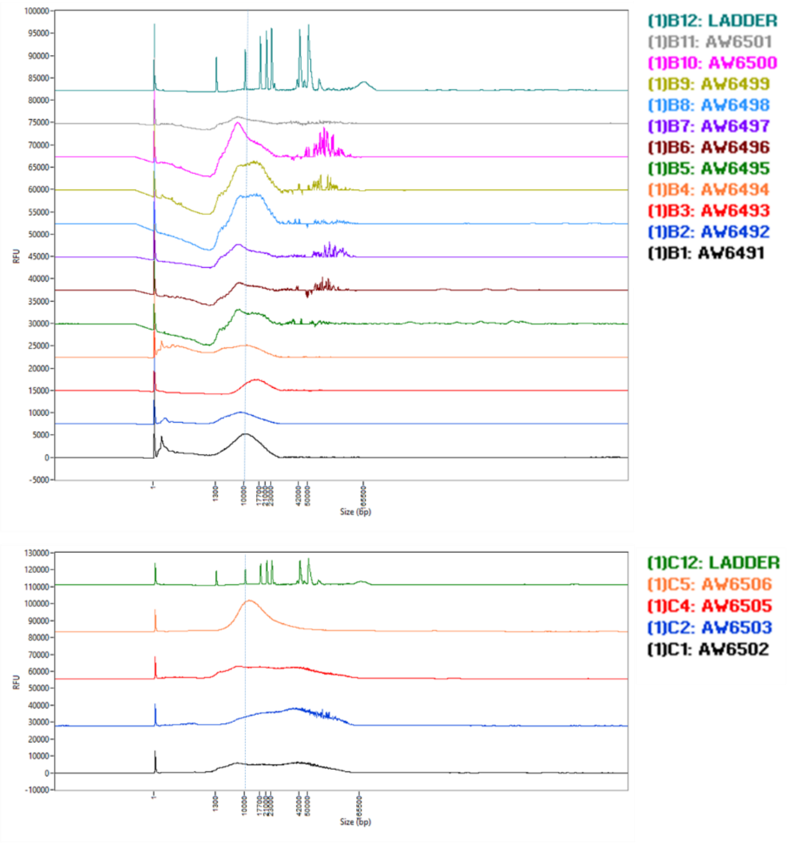

b

Sample ID	DQN
AW6491	3.2
AW6492	3.0
AW6493	9.0
AW6494	1.7
AW6495	6.4
AW6496	7.0
AW6497	5.6
AW6498	6.6
AW6499	6.7
AW6500	5.0
AW6501	5.2
AW6502	6.9
AW6503	8.2
AW6505	6.6
AW6506	6.9

Supplementary Figure 5: Femto Pulse high-resolution sizing of clinical samples gDNA (**a**) Femto Pulse traces of clinical samples gDNA demonstrating the integrity profiles (**b**) DNA quality number (DQN) of clinical samples gDNA. The DQN calculation threshold was specified to 10kb.

Supplementary Note 7

Quality control of clinical samples by lrPCR. Full-length mtDNA amplification product showed SVs in one clinical sample AW6506. We observed two bands representing two mtDNA populations of ~13.5 kb and ~3.5 kb in this sample. Wildtype 16.5 kb mtDNA was not visible on the gel, due to the amplification bias against larger fragments (Supplementary Fig. 6).

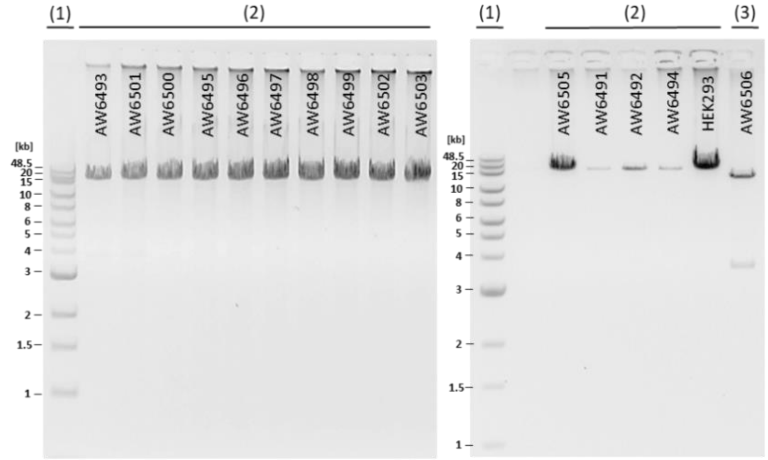

Supplementary Figure 6: Agarose gel of the lrPCR products from the clinical samples. (**1**) molecular weight marker, (**2**) amplicons with 16.5 kb mtDNA, and (**3**) amplicons with two mtDNA SVs in sample AW6506. The lrPCR quality control was performed twice with consistent results. Uncropped gels are provided at the end of the Supplementary Information.

Supplementary Table 1: The crRNA sequences and details of mtDNA target sites.

crRNA name	Cut site and direction	Sequence 5'-3' (PAM)
mt1	chrM: -1006	GTA GTC TAT TTT GTG TCA AC (TGG)
mt3	chrM: +3127	CAA ATT CCT CCC TGT ACG AA (AGG)
mt5	chrM: -5142	TAG TAG TAG GGT CGT GGT GC (TGG)
mt7	chrM: -7144	CGA TGA ATA TGA TAG TGA AA (TGG)
mt9	chrM: +9338	TCC ATA ACG CTC CTC ATA CT (AGG)
mt11	chrM: -11239	TAA ATT AGT GCG ATG AGT AG (GGG)
mt13	chrM: +12767	CCA ACT GTT CAT CGG CTG AG (AGG)
mt15	chrM: +14968	ATC ACT CGA GAC GTA AAT TA (TGG)

Supplementary Table 2: List of clinical samples used in the study.

Sample ID	Type of specimen	Affected gene	Pathogenic variant
AW6491	Oral mucosa	*MT-TL1*	m.3243A>G
AW6492	Oral mucosa	*MT-TL1*	m.3243A>G
AW6494	Urine	*MT-TL1*	m.3243A>G
AW6501	Blood	*MT-TL1*	m.3243A>G
AW6500	Blood	*MT-TL1*	m.3243A>G
AW6495	Blood	*MT-ND5*	m.12781A>G
AW6496	Blood	*MT-ATP6*	m.9185T>C
AW6497	Blood	*MT-ND1*	m.4171C>A
AW6498	Blood	*MT-RNR1*	m.1555A>G
AW6499	Blood	*MT-RNR1*	m.1555A>G
AW6502	Blood	*MT-TL1*	m.3243A>G
AW6503	Blood	*MT-TL1*	m.3243A>G
AW6505	Blood	*MT-TW*	m.5541C>T
AW6493	Muscle	*MT-TK*	m.8344A>G
AW6506	Muscle	mtDNA	deletion

Details of the clinical sample source, affected gene and variant provided by the diagnostic laboratory. There were 9 female and 6 male participants between the ages of 3 – 79; mean age 51.8.

Supplementary References

1. Doench, J. G., Hartenian, E., Graham, D. B., Tothova, Z., Hegde, M., Smith, I., Sullender, M., Ebert, B. L., Xavier, R. J. & Root, D. E. Rational design of highly active sgRNAs for CRISPR-Cas9–mediated gene inactivation. *Nature Biotechnol*. **32**, 1262-1267 (2014).
2. Briner, A. E., Donohoue, P. D., Gomaa, A. A., Selle, K., Slorach, E. M., Nye, C. H., Haurwitz, R. E., Beisel, C. L., May, A. P. & Barrangou, R. Guide RNA Functional Modules Direct Cas9 Activity and Orthogonality. *Mol Cell*. **56**, 333-339 (2014).
3. Brinkman, E. K., Chen, T., de Haas, M., Holland, H. A., Akhtar, W. & van Steensel, B. Kinetics and Fidelity of the Repair of Cas9-Induced Double-Strand DNA Breaks. *Mol cell*. **70**, 801-813 (2018).
4. Sternberg, S. H., Redding, S., Jinek, M., Greene, E. C. & Doudna, J. A. DNA interrogation by the CRISPR RNA-guided endonuclease Cas9. *Nature* **507**, 62-67 (2014).
5. Gilpatrick, T., Lee, I., Graham, J. E., Raimondeau, E., Bowen, R., Heron, A., Downs, B., Sukumar, S., Sedlazeck, F. J. & Timp, W. Targeted nanopore sequencing with Cas9-guided adapter ligation. *Nature Biotechnol*. **38**, 433-438 (2020).

Uncropped Supplementary Fig. 6

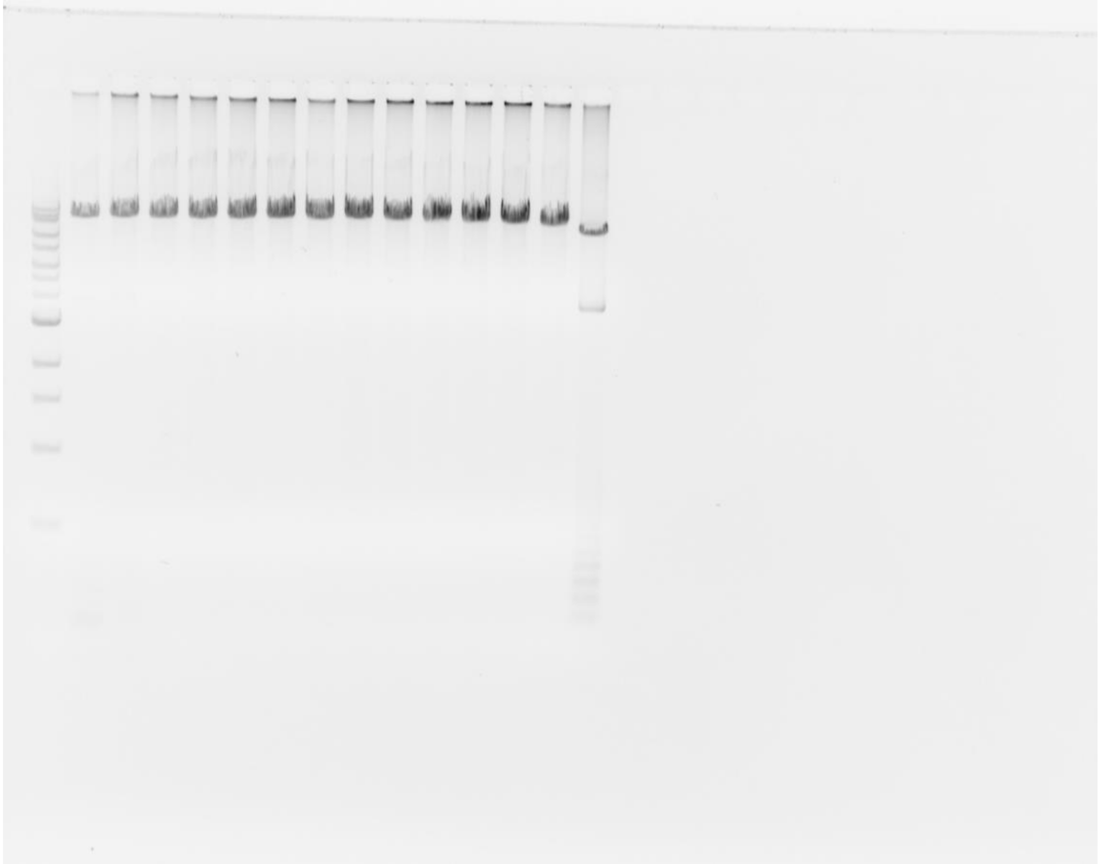

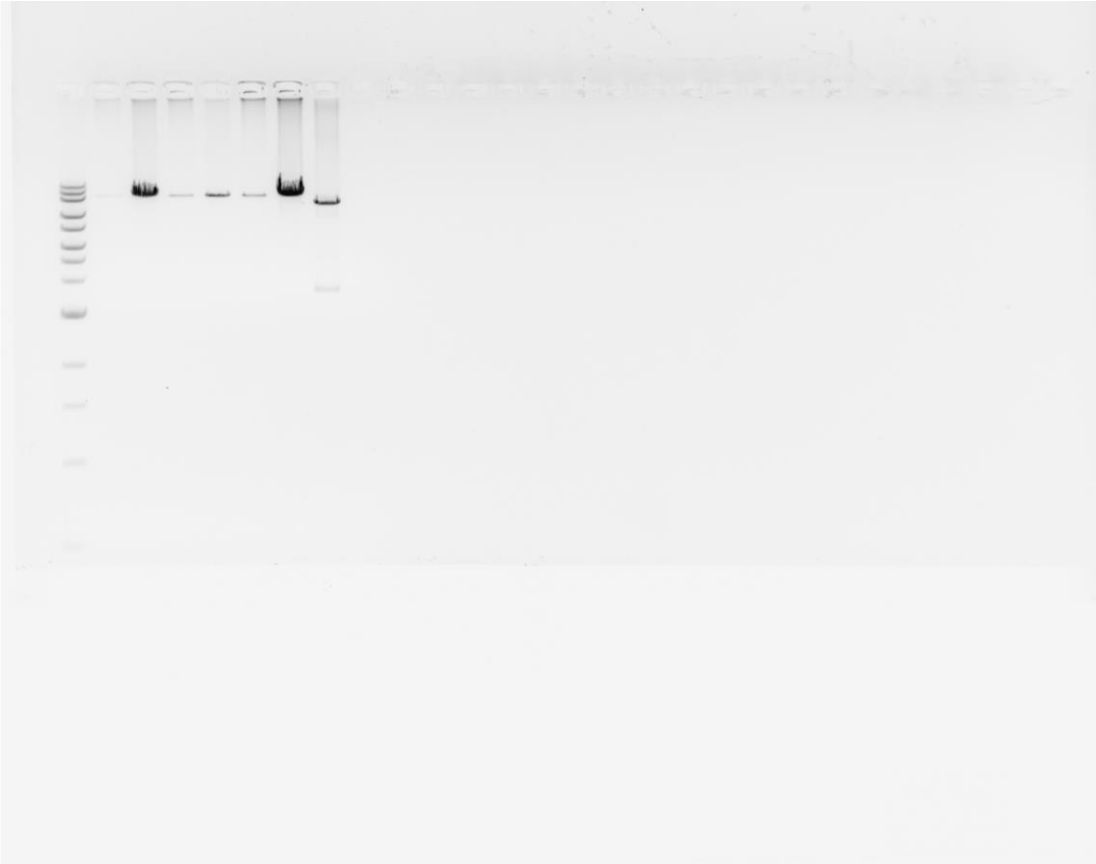

Supplement: Supplementary file 1 — Supplementary Information [file 41467_2022_33530_MOESM1_ESM.pdf]
