# Supplementary material for: A method for multiplexed full-length single-molecule sequencing of the human mitochondrial genome
Source: Nat Commun. 2022 Oct 6;13:5902. doi: 10.1038/s41467-022-33530-3 (PMC9537161; doi:10.1038/s41467-022-33530-3)
Supplement: Supplementary file 3 — Description of Additional Supplementary Files Document [file 41467_2022_33530_MOESM3_ESM.pdf]

Descriptions of Additional Supplementary Files

Supplementary Data 1: Read coverage of the clinical samples demultiplexed with different strategies. All 15 samples were analysed with Cas9-mtDNA-enrichment. In addition, sample AW6506 was amplified by lrPCR and sequenced on ONT. For all sequencing experiments R10.4 flow cells and Q20+ ligation sequencing kit were used.

Supplementary Data 2: Read coverage of Cas9-mtDNA-enrichment sequencing of high integrity gDNA. The coverage after demultiplexing reflects the reliability of RNA-guided Cas9 cut multiplexing. For all sequencing experiments R10.4 flow cells and Q20+ ligation sequencing kit were used.

Supplementary Data 3: Read coverage of Cas9-mtDNA-enrichment sequencing of high integrity gDNA samples after optional gDNA treatment by Exonuclease V.

Supplementary Data 4: Coverage and average divergence per base table of Cas9-mtDNA-enrichment sequencing performed under optimized library preparation conditions. The demultiplexing strategy applied on the sequencing data from different ONT flow cell versions coupled with the Q20+ ligation sequencing kit.

Supplementary Data 5: Variant calling summary of homoplasmic and heteroplasmic sites in ONT and Illumina sequencing data.

Supplementary Data 6: List of haplogroups found, annotation values legend and individual clinical sample annotation results.
