# Supplementary material for: A method for multiplexed full-length single-molecule sequencing of the human mitochondrial genome
Source: Nat Commun. 2022 Oct 6;13:5902. doi: 10.1038/s41467-022-33530-3 (PMC9537161; doi:10.1038/s41467-022-33530-3)
Supplement: Supplementary file 6 — Reporting Summary [file 41467_2022_33530_MOESM6_ESM.pdf]

nature portfolio

Corresponding author(s): Ivo Glynne Gut

Last updated by author(s): Sep 2, 2022

Reporting Summary

Nature Portfolio wishes to improve the reproducibility of the work that we publish. This form provides structure for consistency and transparency in reporting. For further information on Nature Portfolio policies, see our Editorial Policies and the Editorial Policy Checklist.

Statistics

For all statistical analyses, confirm that the following items are present in the figure legend, table legend, main text, or Methods section.

n/a	Confirmed	
☐	☒	The exact sample size (*n*) for each experimental group/condition, given as a discrete number and unit of measurement
☐	☒	A statement on whether measurements were taken from distinct samples or whether the same sample was measured repeatedly
☐	☒	The statistical test(s) used AND whether they are one- or two-sided<br>*Only common tests should be described solely by name; describe more complex techniques in the Methods section.*
☒	☐	A description of all covariates tested
☒	☐	A description of any assumptions or corrections, such as tests of normality and adjustment for multiple comparisons
☐	☒	A full description of the statistical parameters including central tendency (e.g. means) or other basic estimates (e.g. regression coefficient) AND variation (e.g. standard deviation) or associated estimates of uncertainty (e.g. confidence intervals)
☐	☒	For null hypothesis testing, the test statistic (e.g. *F*, *t*, *r*) with confidence intervals, effect sizes, degrees of freedom and *P* value noted<br>*Give P values as exact values whenever suitable.*
☒	☐	For Bayesian analysis, information on the choice of priors and Markov chain Monte Carlo settings
☒	☐	For hierarchical and complex designs, identification of the appropriate level for tests and full reporting of outcomes
☒	☐	Estimates of effect sizes (e.g. Cohen's *d*, Pearson's *r*), indicating how they were calculated

Our web collection on statistics for biologists contains articles on many of the points above.

Software and code

Policy information about availability of computer code

Data collection

For the analysis of short-read data, image analysis, basecalling and quality scoring of the run were processed using the manufacturer's software Real Time Analysis (RTA v3.4.4) and followed by generation of FASTQ sequence files.
For nanopore sequencing the quality metrics of the runs were followed with the MinKNOW software (v.21.05.20-21.10.8). For the analysis of nanopore we used a custom snakemake pipeline55 (https://github.com/marcDabad/q20plus_rebasecall), which involves four different tools. First, the basecalling of the simplex data was performed using Guppy v5.0.16 (https://nanoporetech.com/community). Then, the Duplex Sequencing Tools v0.2.3 (https://github.com/nanoporetech/duplex-tools/) was used to identify and filter the paired duplex reads. Following, the putative duplex reads filtered were high quality basecalled using Guppy Duplex Basecalling beta v0.0.0 (https://nanoporetech.com/community). The last step consisted of merging and removing the redundancy from the two basecalling data results. For this task, we applied an in-house developed tool called fastq_merge. It was noted that (a) the % of duplex reads was very low (~1%) and (b) the base qualities of the reads were wrong with all bases having the same, very high, base quality (61). Two models to perform the basecalling were applied: res_dna_r9.4.1_e8.1_sup_v033.cfg (from the Rerio Database) for the FLO-MIN106D (R9.4.1) flow cell generated data, and dna_r10.4_e8.1_sup.cfg (from Guppy) for the FLO-MIN112 (R10.4) data. Quality control checks were performed with MinIONQC.R56 which uses the final sequencing_summary.txt file.
Alignment of nanopore reads for demultiplexing was performed using minimap2 v2.24-r1122.

Data analysis

Short-read data were processed following the Broad Institute's best practices for variant calling of mitochondrial variants. Briefly, starting from a whole genome alignment, those reads mapping to chrM were extracted and aligned with BWA-MEM to a reference containing only chrM sequence or chrM shifted 8 kb. Variant calling was performed on both alignments with mutect (GATK v.4.1.7.0).
For analysis of the ONT data the baldur software, v1.1.8, ont_demult, v0.3.3, and fastq_merge, v0.3.0 was applied.
Circos plot. To prepare the plots, reads were mapped using the software Minimap2 (2.24). Then reads mapping to chrM were extracted and the coverage was computed for each locus using samtools depth (v. 1.14). The output was parsed to create appropriately formatted files for

input to Circos61 (v. 0.69-9).
To reproduce the results in this manuscript code of the ont_demult (https://github.com/heathsc/ont_demult.git) and baldur (https://github.com/heathsc/baldur.git) are available on github.
Variants were annotated with functional annotations, population frequencies and pathogenicity predictors with SnpEff and SnpSift. gnomAD, MITOMAP and MitoTIP were used as sources of annotation. Relevant information fields of each variant were extracted and converted to a tabular format with bcftools.

For manuscripts utilizing custom algorithms or software that are central to the research but not yet described in published literature, software must be made available to editors and reviewers. We strongly encourage code deposition in a community repository (e.g. GitHub). See the Nature Portfolio guidelines for submitting code & software for further information.

Data

Policy information about availability of data

All manuscripts must include a data availability statement. This statement should provide the following information, where applicable:

- Accession codes, unique identifiers, or web links for publicly available datasets
- A description of any restrictions on data availability
- For clinical datasets or third party data, please ensure that the statement adheres to our policy

The clinical and the cell line sequencing data generated in this study have been deposited in the European Genome-phenome Archive (EGA) under the accession codes EGAS00001006203 (https://ega-archive.org/studies/EGAS00001006203) and EGAS00001006280 (https://ega-archive.org/studies/EGAS00001006280), respectively.

Human research participants

Policy information about studies involving human research participants and Sex and Gender in Research.

Reporting on sex and gender	is now reported in the file Supplementary Data 1, Supplementary Table 2.
Population characteristics	mtDNA variant characterization in the set of 15 clinical samples was performed by the Department of Clinical and Molecular Genetics and Rare Disease at Hospital Universitary Vall d´Hebron. The sex and the age of the subjects are provided in Supplementary Table 2 in Supplementary Data 1.
Recruitment	A set of 15 clinical samples was previously collected by the Department of Clinical and Molecular Genetics and Rare Disease at the University Hospital Vall d´Hebron within a diagnostic workflow for subjects presenting with symptoms related to mitochondrial disease. The 15 samples included in the present work were selected according to the Department of Clinical and Molecular Genetics and Rare Disease at the University Hospital Vall d´Hebron mtDNA status results.
Ethics oversight	The diagnostic procedures for these patients followed the internal protocols of the University Hospital Vall d´Hebron. All subjects gave informed consent approved by the bioethics committee (Clinical Research Ethics Committee (CEIC) of the Vall d'Hebron Hospital; Nº CI/0602/2013) and in accordance with the Declaration of Helsinki. There was no compensation for participating in the study.

Note that full information on the approval of the study protocol must also be provided in the manuscript.

Field-specific reporting

Please select the one below that is the best fit for your research. If you are not sure, read the appropriate sections before making your selection.

☒ Life sciences ☐ Behavioural & social sciences ☐ Ecological, evolutionary & environmental sciences

For a reference copy of the document with all sections, see nature.com/documents/nr-reporting-summary-flat.pdf

Life sciences study design

All studies must disclose on these points even when the disclosure is negative.

Sample size	4 cell lines and 15 clinical samples. The experimental design included both human cell lines and clinical samples. The cell lines allowed multiple experiments to be conducted on the same biological material, permitting the reproducibility and accuracy of the variant calling from ONT sequencing data to be assessed by comparing across multiple ONT sequencing experiments, and by comparing estimates from ONT and Illumina sequencing datasets. These assessments are presented in the Results section. The clinical data samples allowed assessment of the heteroplasmy detection by comparing with the known clinical results for the samples. No formal assessment of sample size was performed.
Data exclusions	No data were excluded from the analyses.
Replication	The cell lines allowed multiple experiments to be conducted on the same gDNA sample, permitting the assessment of reproducibility and accuracy of the variant calling from ONT sequencing data comparing across multiple ONT sequencing experiments, and by comparing estimates from ONT and Illumina sequencing datasets. The same samples were analyzed using different nanopore flowcell types. For the

	clinical samples we used at least two guide RNAs per gDNA sample. Our variant calling results on the clinical samples were compared to the results from the Department of Clinical and Molecular Genetics and Rare Disease at Hospital Universitary Vall d´Hebron post hoc and produced identical results.
Randomization	none for the order of samples and the attribution of the guides to samples with SNV was done randomly. Where two guides per sample were used they were chosen to be on opposite sides of the circular mitochondrial genome.
Blinding	Clinical samples were analyzed blind and results compared to the results of the Department of Clinical and Molecular Genetics and Rare Disease at Hospital Universitary Vall d´Hebron post hoc.

Reporting for specific materials, systems and methods

We require information from authors about some types of materials, experimental systems and methods used in many studies. Here, indicate whether each material, system or method listed is relevant to your study. If you are not sure if a list item applies to your research, read the appropriate section before selecting a response.

Materials & experimental systems

n/a	Involved in the study
☒	☐ Antibodies
☐	☒ Eukaryotic cell lines
☒	☐ Palaeontology and archaeology
☒	☐ Animals and other organisms
☒	☐ Clinical data
☒	☐ Dual use research of concern

Methods

n/a	Involved in the study
☒	☐ ChIP-seq
☒	☐ Flow cytometry
☒	☐ MRI-based neuroimaging

Eukaryotic cell lines

Policy information about cell lines and Sex and Gender in Research

Cell line source(s)	Cell lines HEK293, A549, Capan-2, SH-SY5Y were originally obtained from ATCC
Authentication	No additional authentication was performed on the cell lines
Mycoplasma contamination	Cell lines were not tested for mycoplasma contamination.
Commonly misidentified lines (See ICLAC register)	none
